# Quantitative MRI assessment of joint effusion using T2-relaxometry at 3 Tesla: a feasibility and reproducibility study

**DOI:** 10.1007/s00256-024-04652-0

**Published:** 2024-03-21

**Authors:** Flora H. P. van Leeuwen, Beatrice Lena, Eline D. P. van Bergen, Janoah J. van Klei, Merel A. Timmer, Lize F. D. van Vulpen, Kathelijn Fischer, Pim A. de Jong, Clemens Bos, Wouter Foppen

**Affiliations:** 1 Department of Radiology and Nuclear Medicine, Division of Imaging & Oncology, University Medical Center Utrecht, Utrecht University, HP: E01.132, P.O. Box 85500, 3584 CX Utrecht, The Netherlands; 2 Center for Benign Haematology, Thrombosis and Haemostasis, Van Creveldkliniek, University Medical Center Utrecht, Utrecht University, HP C01.428, P.O. Box 85500, 3584 CX Utrecht, The Netherlands

**Keywords:** Magnetic resonance imaging, Haemarthrosis, Synovial fluid, Haemophilia, Reproducibility of results

## Abstract

**Objective:**

T2-relaxometry could differentiate between physiological and haemorrhagic joint effusion (≥ 5% blood) in vitro. Are quantitative T2-relaxation time measurements of synovial fluid feasible and reproducible in vivo in clinically bleed-free joints of men with haemophilia?

**Materials and methods:**

In this cross-sectional study, we measured T2-relaxation times of synovial fluid in clinically bleed-free ankles, knees or elbows of men with severe haemophilia A using a T2-mapping sequence (duration ≤ 7 min) at 3 Tesla MRI. Manual and circular regions of interest (ROI) were drawn in the synovial fluid of each joint by two independent observers to measure T2-relaxation times. Measurement feasibility was expressed as the success rate of the measurements by both observers. The interobserver and intraobserver reproducibility of the measurements were evaluated by the intraclass correlation coefficient of absolute agreement (ICC) and the limits of agreement (LoA) from Bland Altman analysis.

**Results:**

We evaluated 39 clinically bleed-free joints (11 ankles, 12 knees, 16 elbows) of 39 men (median age, 24 years; range 17–33) with severe haemophilia A. The success rate of the T2-measurements was ≥ 90%. Interobserver reliability was good to excellent (manual ROI: ICC = 0.92, 95% CI 0.76–0.97; circular ROI: ICC = 0.82, 95% CI 0.66–0.91) and interobserver agreement was adequate (manual ROI: LoA = 71 ms; circular ROI: LoA = 146 ms). Intraobserver reliability was good to excellent (manual ROI: ICC = 0.78, 95% CI − 0.06–0.94; circular RO: ICC = 0.99, 95% CI 0.98–0.99) and intraobserver agreement was good (manual ROI: LoA = 63 ms; circular ROI: LoA = 41 ms).

**Conclusion:**

T2-relaxometry of synovial fluid in haemophilia patients is feasible with good interobserver and intraobserver reproducibility.

**Supplementary Information:**

The online version contains supplementary material available at 10.1007/s00256-024-04652-0.

## Introduction

Haemarthrosis can occur after trauma, or spontaneously in bleeding disorders such as von Willebrand disease and haemophilia [1, 2]. Haemarthrosis can induce joint damage, even after brief exposure to a small amount of intra-articular blood [3–6]. Recurrent haemarthroses can lead to irreversible arthropathy [3–5], causing pain and impaired joint function that reduce quality of life [1, 3, 7].

Large traumatic haemarthroses are usually diagnosed based on clinical symptoms as pain, swelling and function loss [7]. Additionally, haemarthrosis is characterized by joint effusion on imaging. The haemorrhagic effusion may have a complex appearance, sedimentation of blood cells may be seen, and fluid–fluid levels may be observed with extensive intra-articular soft tissue damage or fractures (lipohaemarthrosis) [8, 9].

Diagnosing small haemarthroses, haemarthroses without observed trauma, and haemarthrosis in joints with arthropathy can be difficult. Clinical symptoms are not specific to haemarthrosis and also occur in other joint conditions such as arthropathy [10–13]. Joint effusion on imaging is not specific either and may be observed in arthropathy flare-ups and arthritis, as in healthy joints [10, 14–17]. Furthermore, the nature of smaller effusions may be difficult to determine with conventional imaging methods. Conventional T1 and T2 weighted magnetic resonance imaging (MRI) is not sensitive to detecting early small haemarthroses [18, 19]. Therefore, T2*-weighted gradient echo (GRE) sequences are used to visually assess acute haemarthrosis and synovial haemosiderin deposition after haemarthrosis [20]. However, they cannot quantify or may not identify minor or subclinical haemarthroses.

Differentiation between haemarthrosis and other diagnoses is important because they require different treatment [7, 10, 21, 22]. Specifically, people with bleeding disorders need clotting factor replacement therapy to stop haemarthrosis and prevent progression to arthropathy [7].

The current reference standard is joint aspiration, an invasive procedure with a risk of intra-articular infection and bleeding, particularly in people with bleeding disorders [7]. An induced haemarthrosis would result in a false-positive outcome. Therefore, joint aspiration is an imperfect reference standard and a non-invasive alternative would be preferable.

In an in vitro setting, T2-relaxometry MRI can quantitatively differentiate between small volumes of physiological joint effusion and haemorrhagic joint effusion with blood concentrations of ≥ 5% blood [23]. Differentiation was based on differences in T2-relaxation times caused by the T2-shorting effect of iron-containing blood [24]. However, in vivo validation of the experimental T2-relaxometry method is required before its use in patients. Establishing good feasibility and reproducibility of the T2-relaxometry method in bleed-free joints is the first step in in vivo validation.

The primary study objective was to evaluate the feasibility and reproducibility of experimental MRI T2-relaxometry of joint effusion in vivo in clinically bleed-free joints of men with severe haemophilia A. Second, we tested robustness of the T2-measurments and determined normal values for T2-relaxation of synovial fluid without blood.

## Materials and methods

### Study design and population

The MRI T2-maps in this study were obtained as part of the Detecting Subclinical Joint BlEedinG and INflammation in Haemophilia study (BEGIN study). The BEGIN study was approved by the institutional medical ethical review board and all study participants gave written informed consent. This cross-sectional study investigated signs of subclinical bleeding and inflammation in adolescent and adult men with severe haemophilia A between December 2019 and March 2022 [25, 26].

**Fig. 1:**
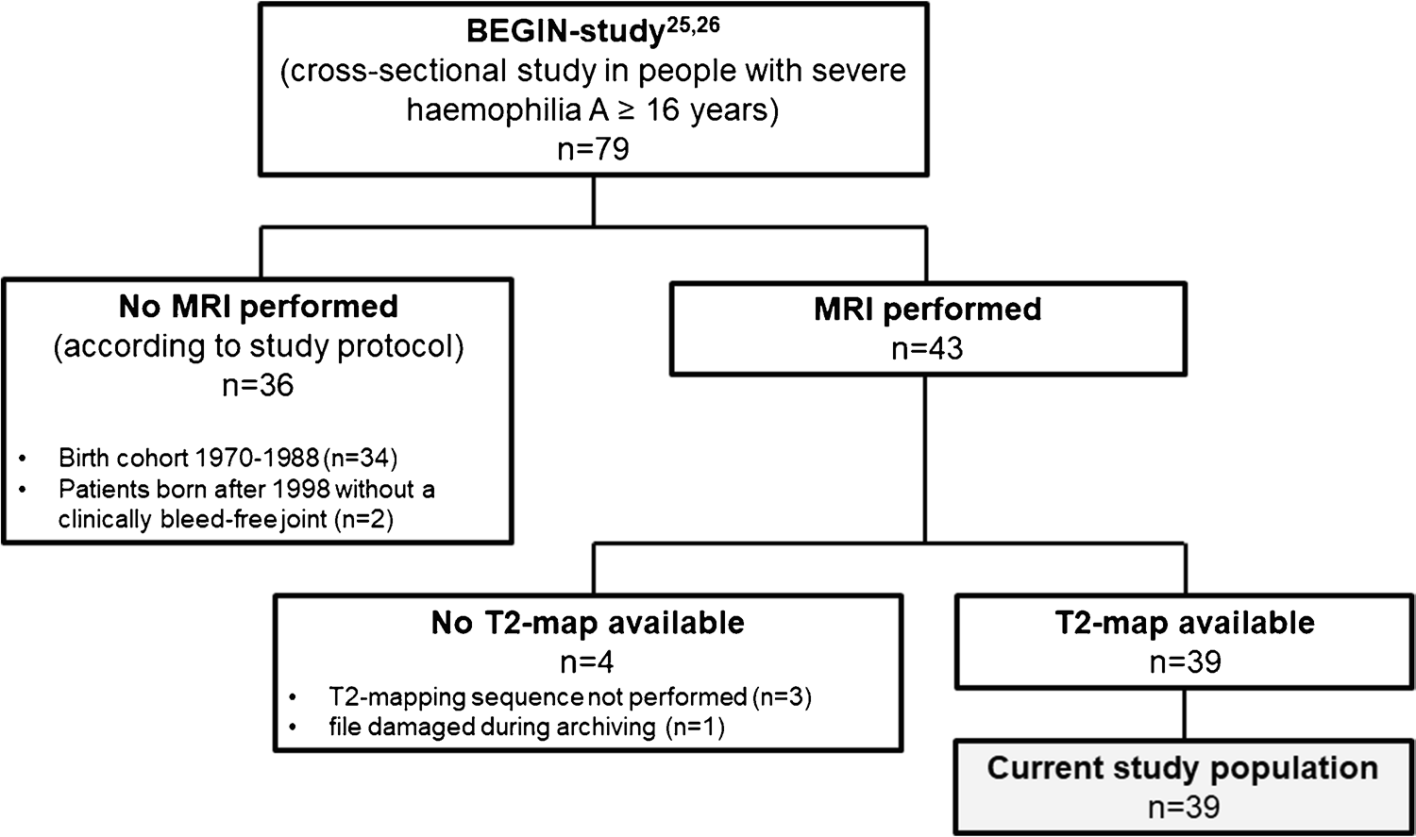
A Flowchart summarizing patient inclusion in the current study

In the BEGIN study, haemophilia patients born after 1988 who received prophylactic treatment reducing the (joint) bleeding risk were screened for a joint (elbow, knee or ankle) without a clinical history of bleeding. In the subgroup of patients with clinically bleed-free joints, one life-long clinically bleed-free joint per patient was examined using MRI. Clinical MRI images were scored for the presence of joint effusion, haemosiderin and synovial hypertrophy by a musculoskeletal radiologist (WF) with > 10 years of experience using the additive International Prophylaxis Study Group (IPSG) MRI score for haemophilic arthropathy [27]. The additive IPSG MRI score grades effusion, synovial hypertrophy and haemosiderin on a scale from 0 to 3 (0 = absent, 1 = minimal, 2 = moderate or 3 = large). The effusion grade was determined using previously described cut-off values [14]. In addition to the clinical MRI images, T2-maps were obtained in 40 joints for use in the current study. Figure 1 shows a flowchart summarizing the inclusion of patients in the current study.

### Magnetic resonance imaging

MRI examinations were performed on three 3 T MRI systems (Philips Achieva, Best, The Netherlands) using joint-specific coils (8-channel small extremity coil for elbows, 16-channel knee coil, 8-channel ankle coil). All three MRI systems were located at the same hospital. Detailed MRI protocols are available in Table 1. MRI protocols included sequences to assess soft tissue (effusion and synovial tissue) and osteochondral (cartilage and bone) pathology, and a GRE sequence to assess haemosiderin deposition according to the IPSG MRI score; these results have been reported elsewhere [25]. In addition, an experimental multi-slice Turbo Spin Echo (TSE) sequence with 32 echoes and SENSitivity Encoding (SENSE) acceleration was included in the imaging protocol for T2 mapping [23]. T2-mapping sequences contained five slices for the knees and three slices for the elbows and ankles. T2-mapping sequence duration was 7 min in the knees and 4 min 12 s in the elbows and ankles.

**Table 1:** Details of the used sequences in the MRI protocols per joint

	Sequence	Plane	TR (ms)	TE (ms)	Flip angle (degrees)	Acceleration (SENSE reduction factor)*	FOV (mm)	Slice thickness (mm)	Slice interval (mm)	In-plane image matrix	Pixel size (mm^2^)	Scanning time (s)
Elbow (8-channel small extremity coil)	T1_TSE	Transversal	657	20	90	-	106*140*82	2.5	2.5	232*170	0.36	140
	PD_TSE	Coronal	2800	25	90	1.3	82*97*120	2.5	2.5	304*178	0.20	163
	T2_ TSE_SPAIR	Sagittal	3639	60	90	-	110*82*120	2.5	2.5	240*165	0.34	226
	T2_ TSE_SPAIR	Coronal	3627	60	90	-	82*95*120	2.5	2.5	172*100	0.67	239
	T2_FFE	Sagittal	516	9.2	25	-	110*82*120	2.5	2.5	244*178	0.31	185
	** T2_Q**	Sagittal	3000	24–1176	90	2	128*13*128	4	4.5	160*156	0.64	252
Knee (16-channel knee coil)	3D_PD_SPAIR	Sagittal	1000	182	90	*P* = 2; *S* = 2	161*161*145	0.8	0.8	180*200	0.65	252
	3D_PD	Sagittal	1000	37	90	*P* = 2; *S* = 2.5	159*161*140	0.52	0.52	268*307	0.27	351
	T2_SPAIR	Sagittal	5949	62	90	-	150*89*150	3	3.3	272*214	0.38	202
	T2_FFE	Sagittal	472	12	20	-	150*150*89	3	3.3	304*243	0.31	232
	**T2_Q**	Transversal	3000	20–980	90	2	128*128*27	5	5.5	160*156	0.64	420
Ankle (8-channel ankle coil)	3D_PD_SPAIR	Coronal	1100	182	90	*P* = 2; *S* = 1	108*120*200	0.8	0.8	252*147	0.64	358
	PD_Dixon	Sagittal	2235	20	90	2	147*82*200	2.5	2.5	476*256	0.22	326
	T2_Dixon	Transversal	7316	100	90	1.7	150*125*175	2.5	2.5	232*136	0.52	307
	T2_FFE	Sagittal	516	9.2	25	-	145*82*200	2.5	2.5	408*235	0.31	190
	**T2_Q**	Sagittal	3000	24–1176	90	2	128*13*128	4	4.5	160*156	0.64	252

*TR* repetition time, *TE* echo time, *SENSE* SENSitivity Encoding, *SENSE reduction factor P, reduction factor S is applicable in 3D sequences only. *FOV* field of view, anterior–posterior*right-left*craniocaudal dimension. *TSE* turbo spin echo, *PD* proton density, *SPAIR*: spectral attenuated inversion recovery, *FFE* fast field echo (Gradient Echo); *T2_Q* multi-slice TSE sequence with 32 equally spaced echo times and SENSE acceleration used for the T2 mapping

### T2 mapping and T2 measurements

T2-maps were obtained by image processing in Matlab version R2021a (The MathWorks, Inc. Natick, MA, USA). The signal intensity (*S*) from the data points was voxel-wise fitted using a Levenberg–Marquardt nonlinear least squares method with the fit function *S* = *S*_0_ (e^−TE/T2^), where *S*_0_ represents the spin density, TE the echo time and T2 the T2-relaxation time [23, 28].

**Fig. 2:**
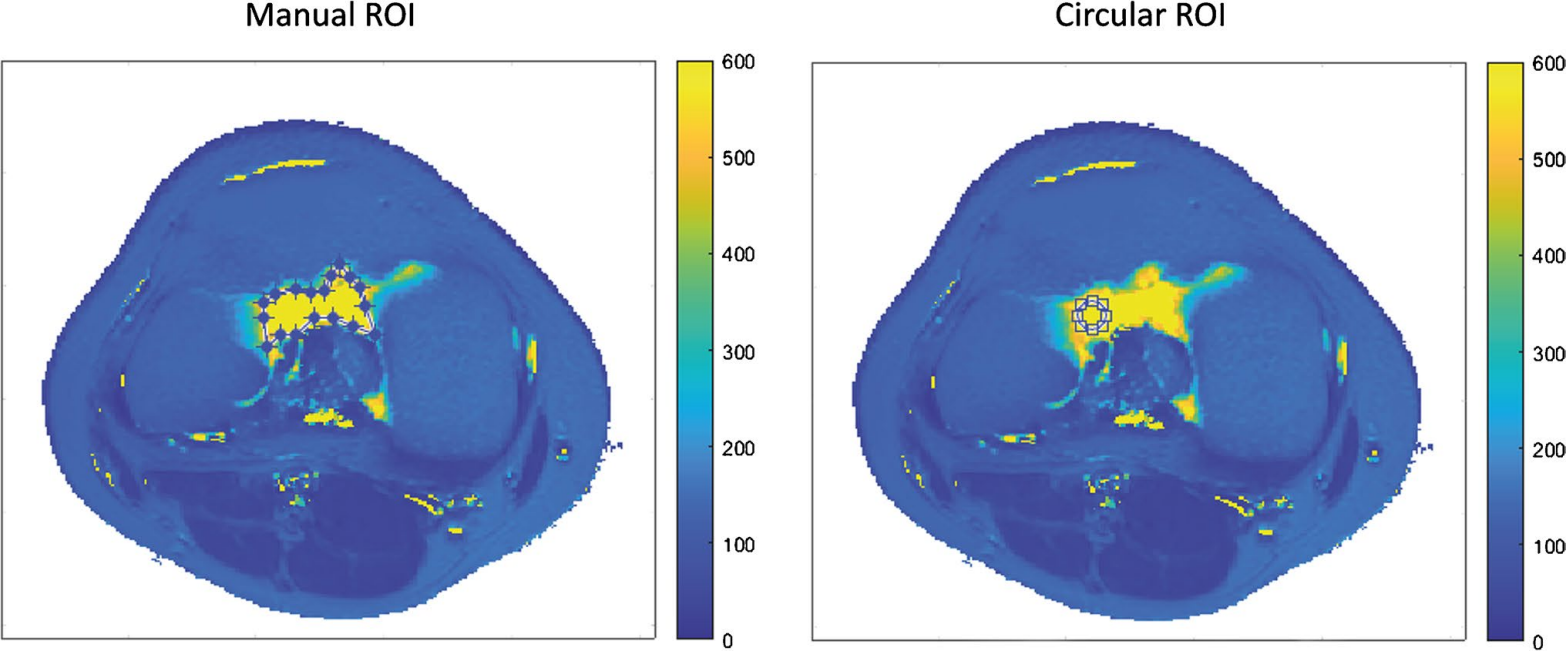
Two examples of a transversal T2-map of a knee with a manual region of interest (ROI) and a circular ROI drawn in the synovial fluid

The T2 measurement of synovial fluid was based on the mean T2-relaxation time of a region of interest (ROI) in the synovial fluid. Two types of ROIs were investigated: manual delineation of visible synovial fluid (manual ROI) and placement of a circular ROI within the synovial fluid (circular ROI) as illustrated in Fig. 2. ROIs were placed on the slice of the T2-map showing the most synovial fluid by two independent observers (FL: medical doctor with 4 months training in clinical radiology; JK: medical student with no radiology experience). One observer (JK) placed the ROIs twice in each joint with an interval of 2 weeks between measurements. Minimising the observer’s learning effect of ROI placement on T2-relaxation times, the second round of ratings (JK2) was used to determine interobserver reproducibility and mean values. To verify ROI placement by the relatively inexperienced observers, a musculoskeletal radiologist (WF) reviewed all manual ROIs for correct placement within the synovial fluid.

### Statistical analysis

Patient and joint characteristics were reported as median values with ranges for continuous variables and as number with frequencies for dichotomous/categorical variables.

To determine the feasibility of T2 measurements in vivo*,* we calculated the proportion of joints where both observers successfully performed the measurement (success rate). Corresponding 95% confidence intervals (CI) were calculated using the Clopper-Pearson Exact method [29].

Interobserver and intraobserver reliability of T2 measurements was assessed using the intraclass correlation coefficient of absolute agreement (ICC) [30]. ICC values < 0.5 indicated poor reliability, values between 0.5 and 0.75 indicated moderate reliability, values between 0.75 and 0.9 indicated good reliability, and values > 0.90 indicated excellent reliability. The interobserver and intraobserver limits of agreement (LoA) were determined using Bland Altman analysis [31]. To evaluate interobserver reliability and agreement, the measurements of the first observer (FL) were compared with the second measurements of the second observer (JK2). The smallest detectable change (SDC) was determined to quantify how large the difference between two measurements must be to be detected by the current measurement procedure. The SDC was calculated as SDC = 1.96*√2*SEM _agreement_. The SEM _agreement_ refers to the standard error of measurement, which equals the square root of the sum of the interobserver/intraobserver variance and the residual variance [30].

Mean T2-relaxation times were calculated from the measurements of the first observer (FL) and the second measurements of the second observer (JK2) and were reported in milliseconds (ms) with standard deviations (sd). The mean T2-relaxation times of the manual and circular ROIs were compared using a paired t-test. To investigate the robustness of the T2-relaxometry method, we investigated whether different joint types and varying amounts of effusion affected the T2 measurements and whether the presence of haemosiderin depositions in the synovial membrane caused significant T2 shortening. Correlations between T2-relaxation time and joint type, the amount of effusion (IPSG MRI scores no/minimal effusion versus moderate/large effusion) and the presence of haemosiderin were determined using multivariate linear regression. All analyses were performed in RStudio version 2022.12.0 + 353 (Posit Software, Boston, MA, USA).

## Results

### Patients

A flowchart summarizing patient inclusion in the current study is available in Fig. 1. T2-maps were obtained for 40 clinically bleed-free joints. One joint was excluded from evaluation because its MRI data was irreparably damaged during file archiving. Therefore, we ultimately measured T2-relaxation time of synovial fluid in 39 clinically bleed-free joints of 39 patients. All 39 patients had severe haemophilia A and were all male because of the recessive X-linked nature of haemophilia. They all received prophylactic treatment reducing the risk of spontaneous and traumatic (joint) bleeding. The median age was 24 years (range 17–33). Joint characteristics of the evaluated joints are available in Table 2. The 39 joints evaluated included 16 elbows (41%), 12 knees (31%) and 11 ankles (28%). None had a history of overt haemarthrosis. However, 6/39 joints (15%) had haemosiderin deposits in the synovium, indicating previous subclinical bleeding [25]. Concomitant synovial hypertrophy was observed in one ankle with haemosiderin deposits. A physiological amount of synovial fluid (no effusion) was observed in 19/39 joints (49%). There was minimal effusion in 12 joints (31%), moderate effusion in 7 joints (18%), and large effusion in 1 joint (3%).

**Table 2:** Joint characteristics

	Total (*n* = 39); *n* (%)	Elbows (*n* = 16); *n* (%)	Knees (*n* = 12); *n* (%)	Ankles (*n* = 11); *n* (%)
Effusion*				
Absent	19 (49%)	13 (81%)	3 (25%)	3 (27%)
Minimal	12 (31%)	3 (19%)	3 (25%)	6 (55%)
Moderate	7 (18%)	0 (0%)	6 (50%)	1 (9%)
Large	1 (3%)	0 (0%)	0 (0%)	1 (9%)
Haemosiderin present	6 (15%)	1 (6%)	0 (0%)	5 (45%)
Synovial hypertrophy present	1 (3%)	0 (0%)	0 (0%)	1 (9%)

*Scored according to the additive IPSG MRI score [27]. Percentages might not add up to 100% due to rounding

### Feasibility

For all 39 joints, correct placement of the manual ROIs within the synovial fluid was verified by a musculoskeletal radiologist (WF), no ROIs required editing. The success rate of the T2-relaxation measurements was 100% in joints with moderate or large effusion (*n* = 8/8, CI 69–100%). In joints with no or minimal effusion, the success rate was 87% using the manual ROI (*n* = 27/31, CI 73–95%) and 94% using the circular ROI (*n* = 29/31, CI 81–99%). Unsuccessful measurements were due to the absence of joint effusion (*n* = 1) or minimal joint effusion (*n* = 3), which hampered placing the ROI.

### Reliability

The interobserver and intraobserver reliability results are summarized in Table 3. Interobserver reliability of the T2 measurements was good to excellent (manual ROI ICC = 0.92, CI 0.76–0.97; circular ROI ICC = 0.82, CI 0.66–0.91). Intraobserver reliability was good to excellent (manual ROI ICC = 0.78, CI − 0.06–0.94; circular ROI ICC = 0.99, CI 0.98–0.99).

**Table 3:** Inter- and intraobserver reproducibility of T2 relaxation measurements

	Interobserver reproducibility			Intraobserver reproducibility		
	Reliability	Agreement		Reliability	Agreement	
	ICC (CI)	LoA	SDC	ICC (CI)	LoA	SDC
Manual ROI	0.92 (0.76–0.97)	71 ms	84 ms	0.78 (− 0.06–0.94)	63 ms	154 ms
Circular ROI	0.82 (0.66–0.91)	146 ms	155 ms	0.99 (0.98–0.99)	41 ms	40 ms

*ROI* region of interest, *ICC* intraclass correlation coefficient of absolute agreement, *CI* 95% confidence interval, *LoA* limit of agreement, *SDC* smallest detectable change

### Agreement

**Fig. 3:**
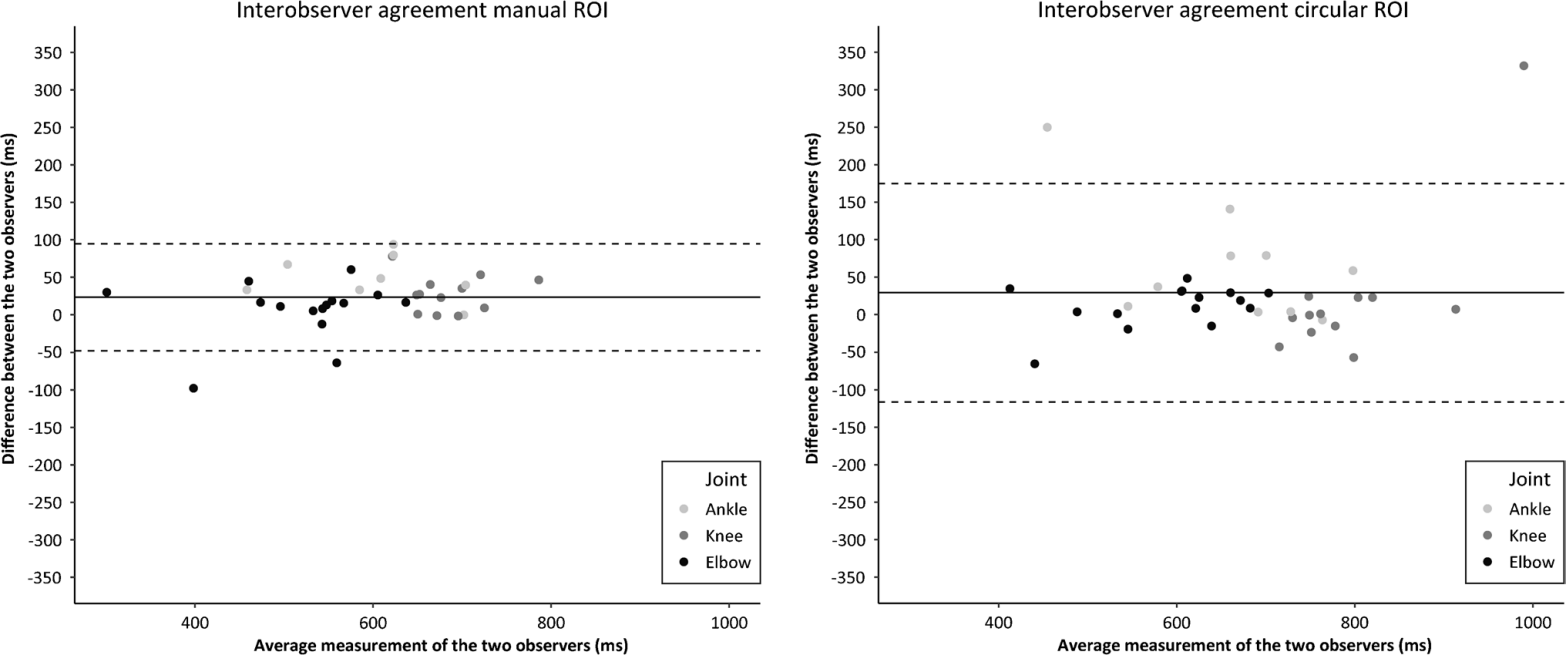
Bland–Altman plots regarding the interobserver agreement of T2-relaxation measurements for the manual and circular regions of interest (ROI)

**Fig. 4:**
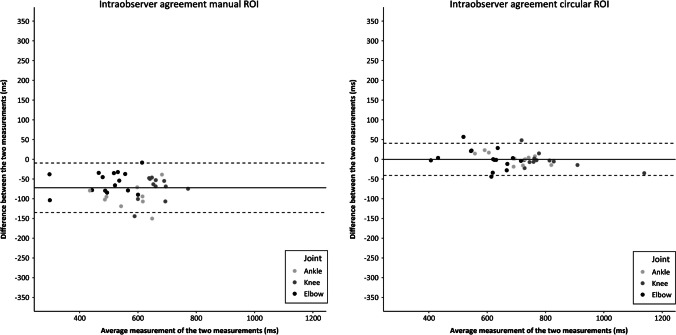
Bland–Altman plots regarding the intraobserver agreement of T2-relaxation measurements for the manual and circular regions of interest (ROI)

The interobserver and intraobserver agreement results are summarized in Table 3. Figures 3 and 4 show the Bland–Altman plots of interobserver and intraobserver agreement.

Interobserver agreement was adequate (manual ROI LoA = 71 ms; circular ROI LoA = 146 ms), without large systematic interobserver differences (manual ROI mean difference = 23 ms; circular ROI mean difference = 29 ms). The SDC based on the interobserver variance ranged from 84 to 155 ms.

Intraobserver agreement was good (manual ROI LoA = 63 ms; circular ROI LoA = 41 ms). A systematic intraobserver difference (mean difference − 72 ms) was observed for the manual ROI measurements. The second measurements of the second observer (JK2) were systematically lower than the first measurements (JK1) which might indicate a slight learning effect after minimal experience with the T2 measurements. For the circular ROI method, there was no systematic intraobserver difference (mean difference 0 ms). The SDC based on the intraobserver variance ranged from 154 to 40 ms.

### T2-relaxation times

**Fig. 5:**
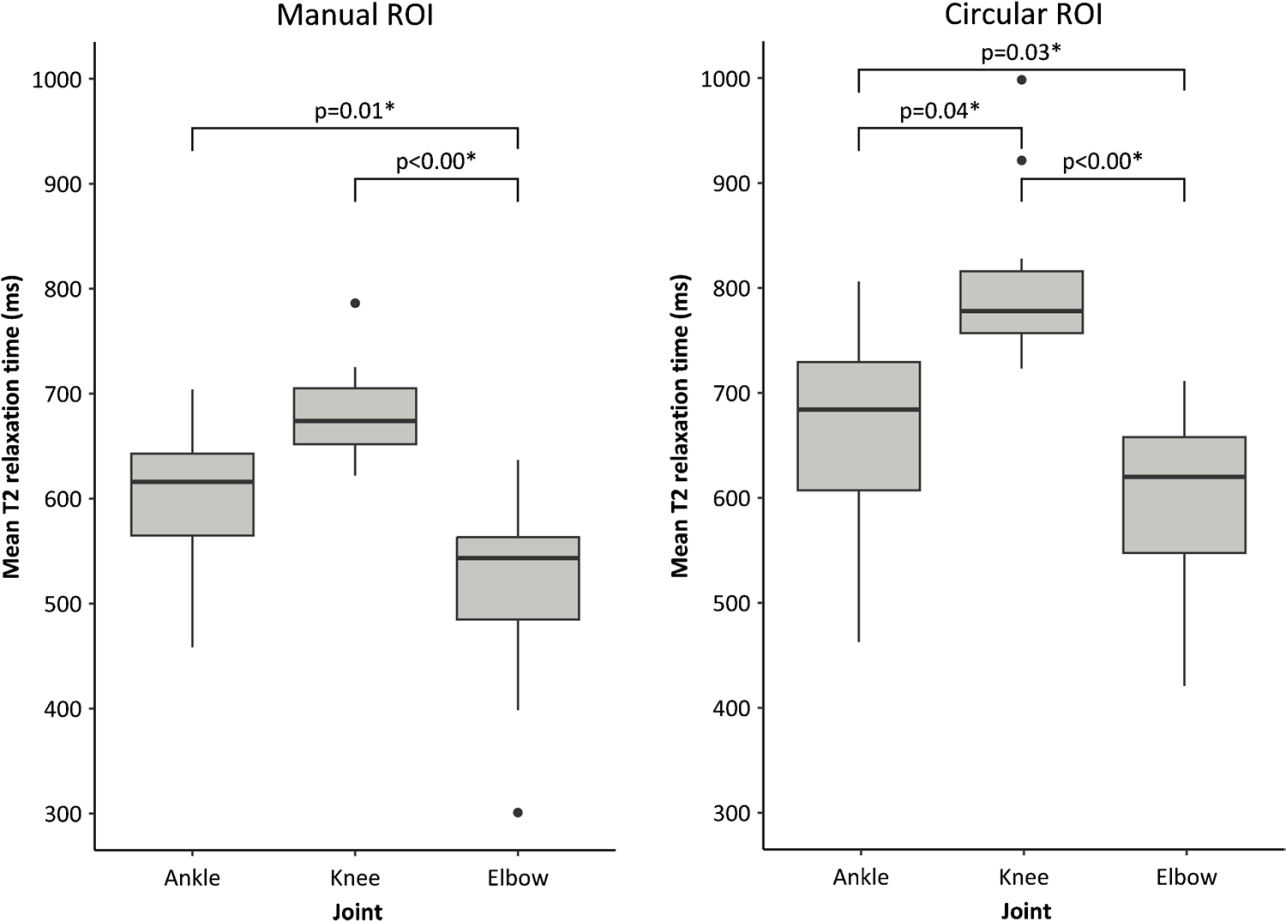
Boxplots showing mean T2-relaxtion times measured with manual and circular regions of interest (ROI) in the different joint types. Asterisk (*) means significant *p*-values from multivariate linear regression adjusted for amount of effusion and presence of haemosiderin deposits in the synovial membrane. The central thick horizontal lines represent the median values, the boxes contain the 1st to 3rd quartiles. The end of the vertical line represents the minimum and maximum values, excluding outliers. Outliers are shown as dots

Mean T2-relaxation times of the synovial fluid are available in Table 4. Results of the multivariate linear regression on the correlations between T2-relaxation time and joint type, the amount of effusion, and the presence of haemosiderin are available in Supplementary Table 1. Circular ROI measurements of T2 relaxation were higher than manual ROI measurements (*p* < 0.00). Joint type (elbow, knee, ankle) significantly affected T2 measurements, even when corrected for the amount of effusion in the joint and presence of haemosiderin deposits in the synovial membrane. Figure 5 shows boxplots of T2-relaxation time for different joints. For manual ROIs, T2-relaxation times in the elbows were significantly shorter than T2-relaxation times in the ankles and knees. For circular ROIs, T2-relaxation times were significantly different for all types (*p* ≤ 0.04). T2-relaxation times were shortest in the elbows, followed by the ankles and knees.

**Table 4:** Mean T2 relaxation times of synovial fluid per joint

T2 relaxation times	Total (*n* = 39)		Elbows (*n* = 16)		Knees (*n* = 12)		Ankles (*n* = 11)	
	Mean (± sd)	sd (± sd)	Mean (± sd)	sd (± sd)	Mean (± sd)	sd (± sd)	Mean (± sd)	sd (± sd)
Manual ROI (ms)	595 (± 102)	92 (± 28)	520 (± 85)	74 (± 20)	684 (± 44)	109 (± 23)	601 (± 86)	100 (± 32)
Circular ROI (ms)	675 (± 125)	45 (± 29)	590 (± 88)	33 (± 14)	797 (± 80)	53 (± 34)	658 (± 105)	53 (± 35)

*ms* milliseconds, *ROI* region of interest, *sd* standard deviation

Presence of haemosiderin showed a non-significant T2 shortening compared to the absence of haemosiderin (manual ROI − 57 ms, *p* = 0.20; circular ROI − 74 ms, *p* = 0.13). T2-relaxation times were not correlated with the amount of effusion (manual ROI *p* = 0.79; circular ROI *p* = 0.97).

## Discussion

We evaluated the feasibility and reproducibility of MRI T2-relaxometry of non-haemorrhagic joint effusion in vivo using MRI data from 39 clinically bleed-free joints in men with severe haemophilia A. T2-relaxometry measurements were feasible in all joints with moderate or large effusion and ≥ 90% of joints with no or minimal effusion. Both manual and circular ROIs showed good–excellent inter- and intraobserver reliability and agreement. Additionally, we obtained normal values of T2-relaxation time of synovial fluid without blood. Mean T2-relaxation times of synovial fluid were 595 ms (± 102) when effusion was manually delineated and 675 ms (± 125) when a circular ROI was placed within the effusion. T2-relaxation times appeared to vary among different joint types.

Differences in T2-relaxation times for different joints may be due to anatomical difference between joints. For example, knees are larger and have a physiologically larger volume of synovial fluid. This makes it easier to place a ROI in the synovial fluid without including surrounding tissue, making measurements less susceptible to partial volume effects. In addition, it is likely that the volume of the ROI will influence the precision of the measurement and hence the standard deviation. Because of significant differences in T2-relaxation times between joints, joint-specific normal values for synovial fluid T2-relaxation times should be used.

### Comparison to previous publications

T2-relaxation times in the current study are consistent with reported T2-relaxation times of synovial fluid in both in vitro and in vivo studies. A previous in vitro study reported a mean T2-relaxation time of 592 ms (± 13) for synovial fluid at 3 Tesla MRI [23], comparable to the mean T2-relaxation times measured with manual ROI (595 ms ± 102) and circular ROI (675 ms ± 125) in the current in vivo study. Two studies measuring T2-relaxation times of synovial fluid in the knees of healthy volunteers reported T2-relaxation times of 767 ms (± 49) [32] and 653 ms (± 113) [33] at 3 Tesla MRI. These are similar to the 684 ms (± 44) in the knees using manual ROI and the 797 ms (± 80) in knees using circular ROI in the current study.

We acknowledge the important work of previous investigators showing conventional T1 and T2 weighted MRI could not qualitatively discriminate physiological synovial fluid or haemorrhagic joint effusion. They report preliminary evidence (mainly ex vivo) that ultrasound is sensitive to small amounts of intra-articular blood [18, 19]. Moreover, GRE sequences are used for visual qualitative differentiation of haemarthrosis in current clinical practice [20]. However, T2-relaxometry may potentially be of interest for detecting small subclinical haemarthrosis in research settings. The previous in vitro results [23] combined with good feasibility and reproducibility of the experimental T2-measurements in vivo suggest the experimental T2-relaxometry method allows quantitative differentiation of physiological synovial fluid from haemorrhagic joint effusion (with low blood concentrations).

### Future research and applications

We demonstrated good feasibility and reproducibility of T2-relaxometry in bleed-free joints. T2 measurements failed in a few joints with no or minimal effusion. However, T2-relaxometry will only be relevant in joints suspected of bleeding. This suspicion requires at least some effusion, increasing the probability of a successful measurement. T2 measurements were not significantly affected by haemosiderin deposits. Therefore, T2 measurements remain useful in joints with a history of bleeding.

The 3-slice T2-relaxometry sequence of 4 min 12 s may be feasible in clinical or research settings. The 5-slice sequence for the knee may be shorted by scanning 3 slices at the effusion site only.

Demonstrating good feasibility and reproducibility was the first step in validating the T2-relaxometry method for differentiation between physiological and haemorrhagic joint effusion in vivo. The next step would be confirming T2 shortening of haemorrhagic joint effusion in vivo*.* This requires a future study including patients with (suspected) haemarthrosis. After confirmation of T2 shortening of haemorrhagic effusion in vivo, T2-relaxometry can serve as a non-invasive alternative to joint aspiration for the diagnosis of haemarthrosis. The T2-relaxation times obtained in the current study can then be used as normal values for synovial fluid without blood. Furthermore, it is known that T2-relaxometry results depend on the magnetic field strength as shown by a previous in vitro study [23]. Future studies are needed to evaluate the test–retest reproducibility of the method and to explore the potential variability of the T2-relaxometry when the measurements are performed in a different institution and/or on different 3 T MR systems from different vendors.

### Limitations

Measuring T2-relaxation times of non-haemorrhagic synovial fluid in men with haemophilia could be seen as a limitation, as they do not reflect the healthy general population. However, there is no (patho)physiological indication to assume differences in synovial fluid between our study patients and healthy volunteers. In addition, haemophilia patients are a large part of the target population for non-invasive diagnosis of haemarthrosis. Therefore, it is logical to perform the study in this population.

Lack of joint aspiration as a reference standard for the diagnosis of joint bleeds can be considered another limitation. However, as the patients had no symptoms or history of bleeding in the examined joint, likelihood of haemarthrosis prior to or during MRI is negligible. Furthermore, a false-positive aspiration due to induced haemarthrosis is possible in people with a bleeding disorder and joint aspiration has the risk of inducing intra-articular infection [7]. Therefore, in addition to the ethical concerns, performing joint aspiration in this cohort would have been contraindicated.

ROI placement by relatively inexperienced observers could be seen as a third limitation. However, reliability and agreement were good to excellent despite the lack of experience and a musculoskeletal radiologist verified accurate ROI placement for all joints. Therefore, no prior knowledge or extensive training seems required to perform measurements reliably. Circular ROIs are probably easiest to implement, as circles are easy and quick to place while maintaining good reproducibility. Manual ROIs showed better interrater reproducibility. However, manual delineation is more time-consuming and has a higher risk of interference from the surrounding (haemosiderotic) synovial membrane. Furthermore, its slightly worse intraobserver reproducibility showed potential dependency on observer’s experience, indicating automated joint effusion segmentation can improve the value of manual ROI.

Finally, heterogeneity in joint types, effusion grades and hemosiderin presence in our study population could have confounded results. Yet, only joint type affected T2 measurements significantly. Therefore, we reported joint-specific T2-relaxation times of synovial fluid.

Quantitative MRI T2-relaxometry can be used to measure T2-relaxation times of joint effusions in patients with good inter- and intraobserver reliability and agreement. Both manual delineation of the effusion and placement of a circular ROI within the effusion are feasible and reproducible. Mean T2-relaxation times obtained in this study may be used as reference values for synovial fluid in the elbows, knees and ankles without bleeding.

## Supplementary Information

Below is the link to the electronic supplementary material.

Supplementary file1 (DOCX 16 KB)

## Data Availability

Data is available from the senior author (Dr. W. Foppen, w.foppen@umcutrecht.nl) upon request.
