# Supplementary material for: Quantitative MRI assessment of joint effusion using T2-relaxometry at 3 Tesla: a feasibility and reproducibility study
Source: Skeletal Radiol. 2024 Mar 21;53(11):2389–98. doi: 10.1007/s00256-024-04652-0 (PMC11410847; doi:10.1007/s00256-024-04652-0)
Supplement: Supplementary file 1 — Supplementary file1 (DOCX 16 KB) [file 256_2024_4652_MOESM1_ESM.docx]

| **Supplementary Table 1.** Results of multivariate linear regression models showing the influence of joint type on T2-relaxation times, adjusted for the amount of effusion and presence of haemosiderin deposits in the synovial membrane (T2-relaxation time =f(joint type\|effusion + haemosiderin)). | | | | |
| --- | --- | --- | --- | --- |
|  | **Manual ROI** | | **Circular ROI** | |
|  | β | p-value | β | p-value |
| **Joint type** |  |  |  |  |
| Elbow (reference) | - | - | - | - |
| Knee | 156 | <0.000* | 201 | <0.000* |
| Ankle | 97 | 0.012* | 100 | 0.027* |
|  |  |  |  |  |
| Ankle (reference) |  |  |  |  |
| Elbow | -97 | 0.012* | -100 | 0.027* |
| Knee | 60 | 0.136 | 101 | 0.040* |
| **Moderate/large effusion**^¶^ | 9 | 0.79 | 2 | 0.97 |
| **Haemosiderin present** | -57 | 0.20 | -74 | 0.13 |
| ROI: Region of interest; ^¶^scored according to the effusion item of the International Prophylaxis Study Group (IPSG) magnetic resonance imaging (MRI) score. *p-value <0.05 | | | | |
